# Untreated sewage outfalls do not promote *Trichodesmium* blooms in the coasts of the Canary Islands

**DOI:** 10.1038/s41598-020-75447-1

**Published:** 2020-10-27

**Authors:** Mar Benavides, Javier Arístegui

**Affiliations:** 1 Aix Marseille Univ, Université de Toulon, CNRS, IRD, MIO UM 110, 13288 Marseille, France; 2 Instituto de Oceanografía y Cambio Global, Universidad de Las Palmas de Gran Canaria, Las Palmas, Spain

**Keywords:** Biogeochemistry, Ocean sciences

## Abstract

During the summer of 2017, recurrent extensive blooms of the diazotrophic cyanobacterium *Trichodesmium* invaded the beaches and coastal waters of the Canary Islands, causing great social alarm. Some local media and public sectors ascribed, without any strong scientific evidence, the origin and reactivation of these blooms to untreated sewage outfalls distributed along the coasts. In order to test whether sewage outfalls could have any influence on the metabolic activity of *Trichodesmium*, we performed ^13^C and ^15^N_2_ uptake experiments with colonies experiencing three different bloom development stages, incubated both with clear seawater and sewage water from an outfall south of Gran Canaria island. Our results showed that sewage outfalls did not promote any increase in dinitrogen (N_2_) fixation in *Trichodesmium*, supporting the hypothesis that decaying blooms were generated offshore and transported shoreward by local currents and winds, accumulating mostly leeward of the islands. The combination of unusually warm seawater temperatures, enhanced and sustained stratification of the upper water column and recurrent dust deposition events would have favored the development of the *Trichodesmium* blooms, which lasted for at least four months.

## Introduction

*Trichodesmium* is a colonial filamentous cyanobacterium capable of atmospheric dinitrogen (N_2_) fixation. It abounds in subtropical and tropical oceanic waters, often forming blooms large enough to be detected by satellite images^1^, rendering a source of fixed nitrogen that fuels primary production significantly^2^. Its growth may be limited however by the availability of iron^3^ and phosphorus^4–6^. In the open waters of the subtropical North Atlantic Ocean, the seasonal meridional shift of the North African dipole controls the deposition of iron-rich Saharan dust on surface waters^7^. Dust events are known to enhance the proliferation and diazotrophic activity of *Trichodesmium* leading to the formation of massive blooms^8–11^, with colonies adopting three-dimensional shapes known as "puffs" or “tufts”. Their organic matter coating as well as the microbial epibiont community provide *Trichodesmium* colonies the ability to degrade atmospheric dust particles to obtain the iron they contain^12,13^, which is likely not possible for free single *Trichodesmium* filaments^14^.

Although it is not clear what mechanism, or combination of mechanisms, triggers the development of *Trichodesmium* blooms, warm (> 20 °C) and stratified waters as well as the availability of phosphorus and iron, are essential requirements to sustain extensive blooms^15^. Under such conditions, *Trichodesmium* develops swiftly, fixing high amounts of carbon and nitrogen^16^. This intense activity may exhaust limiting nutrients, forcing *Trichodesmium* filaments to release exopolymeric substances while aggregating and forming dense colonies^17^. This strategy optimizes nutrient uptake and repartition of resources between filaments of a same colony^17,18^. When all limiting resources are depleted, *Trichodesmium* cells induce apoptosis, also referred to as programmed cell death (PCD)^19^, a process that can make *Trichodesmium* blooms almost disappear in a matter of hours to days^20^.

Traditionally considered an autotroph^16^, *Trichodesmium* is now known to use dissolved organic matter (DOM) molecules which may provide it with alternative nutrient resources when their inorganic forms are unavailable or their use becomes too energetically demanding. *Trichodesmium* is able to use dissolved organic phosphorus (DOP) forms including phosphoesters and phosphonates when inorganic phosphate availability is low^5,6^. The RuBisCo enzyme of *Trichodesmium* has a low affinity for CO_2_^21^, which is thought to explain its enhanced carbon fixation when exposed to high CO_2_ levels and/or lower pH^22,23^. The inefficiency of RuBisCo may drive *Trichodesmium* to use DOM to meet its carbon needs. For example, natural *Trichodesmium* colonies from the Southwest Pacific Ocean have been observed to obtain as much carbon via amino acids as via CO_2_^24^. Cultures of *Trichodesmium* have been reported to grow faster on combined nitrogen forms such as urea than on N_2_^25^. More recent laboratory experiments have shown that when grown under limiting concentrations of iron and/or phosphorus, *Trichodesmium* downregulates N_2_ fixation and obtains nitrogen from trimethylamine or ammonium^26^. Collectively, these studies suggest that *Trichodesmium* adopts a mixotrophic nutrition mode when its basic metabolic activities are reduced due to environmental stress or a limitation of resources. Mixotrophy thus confers *Trichodesmium* metabolic plasticity and adaptation to dynamic environmental nutrient scenarios.

**Figure 1:**
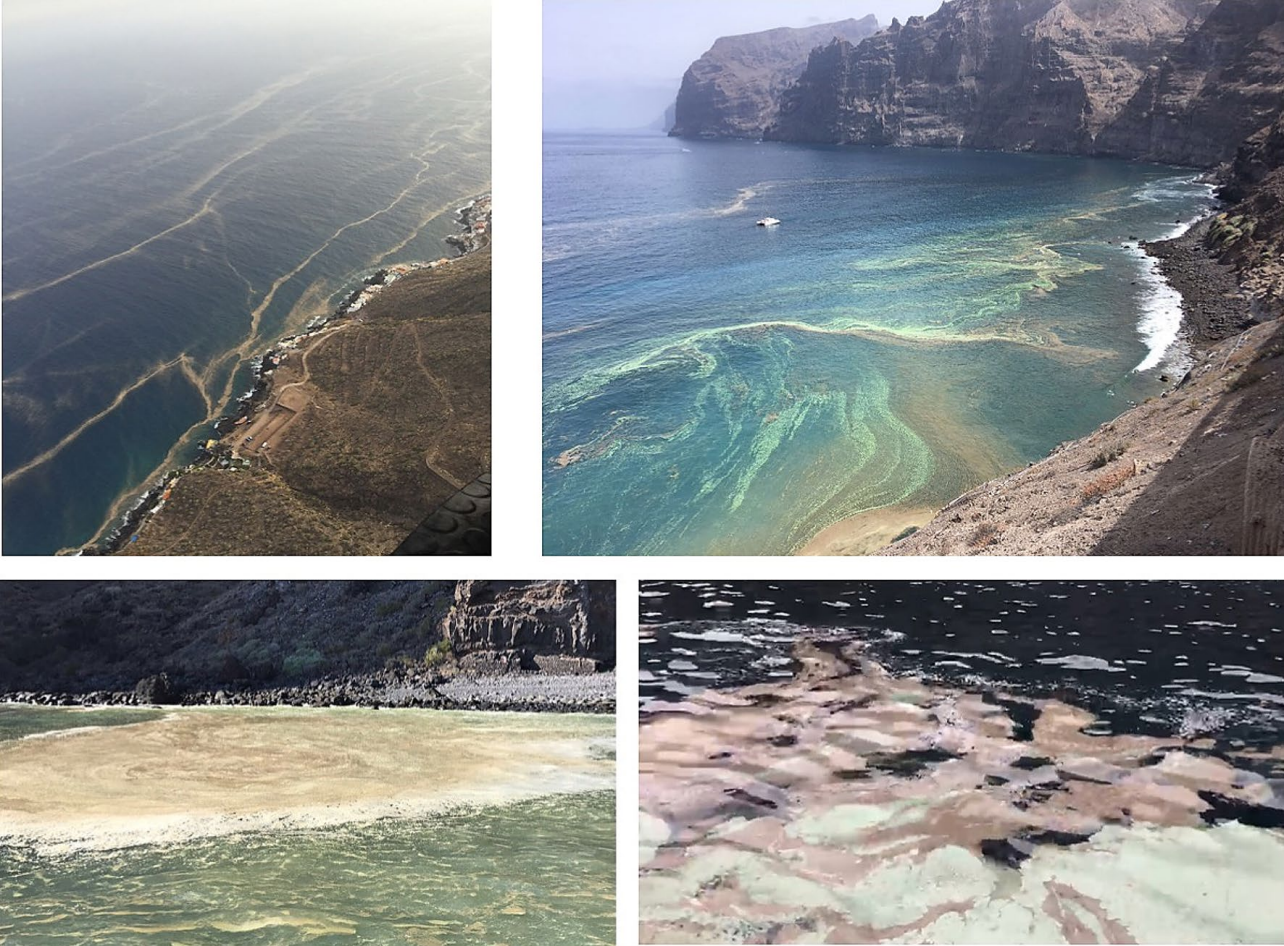
*Trichodesmium* blooms in the coastal waters of the Canary Islands in the summer of 2017. The foamy and cyan-reddish waters are indicative of decaying blooms (i.e. colonies in PCD-mode).

**Figure 2:**
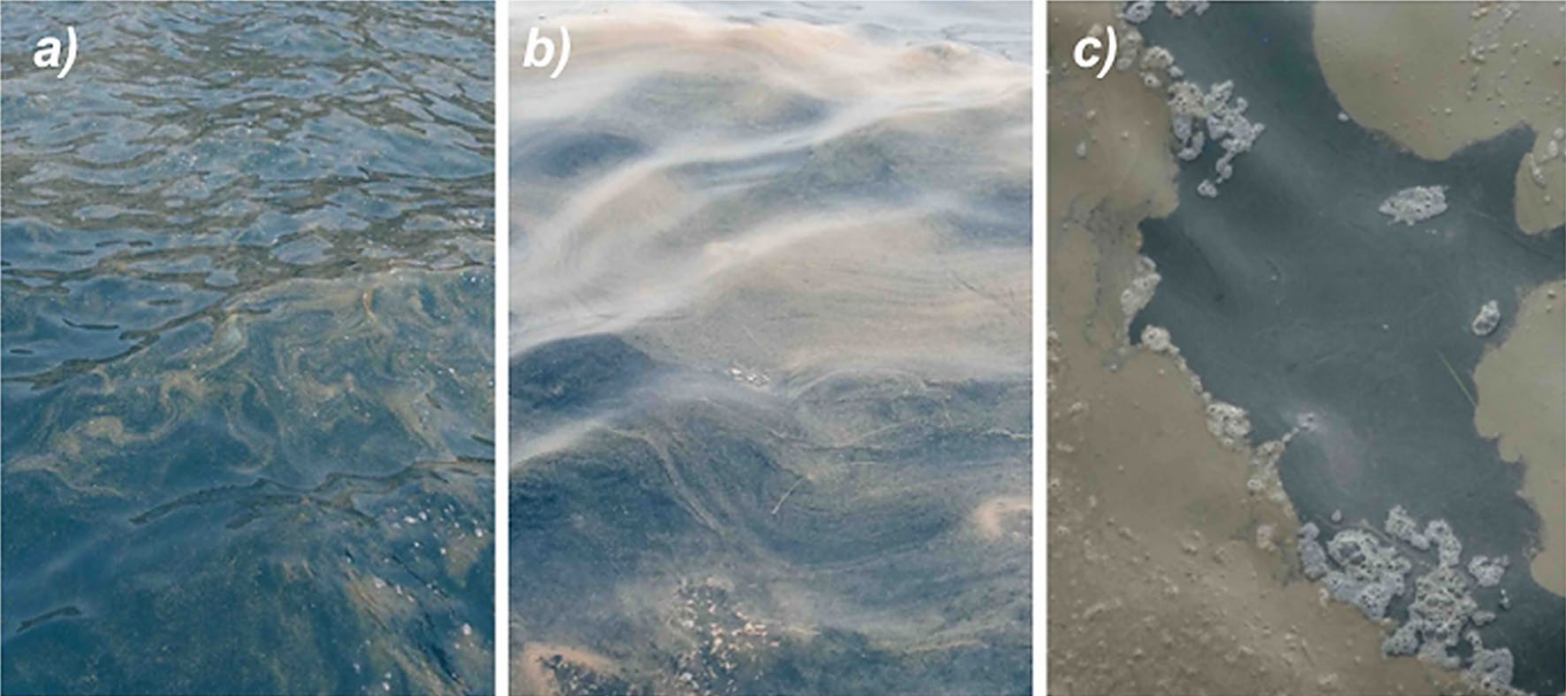
*Trichodesmium* blooms in the coastal waters of the Canary Islands under three development stages: (**a**) sparse, (**b**) slick, (**c**) PCD-like.

Throughout the summer of 2017, recurrent blooms of *Trichodesmium* were observed in coastal and offshore waters around the Canary Islands^27^. These blooms appeared first in the western side of the archipelago, coinciding with dense Saharan dust deposition events, enhanced water column stratification and seawater temperatures > 23 °C, later spreading to the eastern side of the archipelago, when those conditions extended eastwards^27^. The massive accumulations of *Trichodesmium* along the coasts of the Canary Islands (see Fig. 1) caused great social alarm, and were attributed by some media and public sectors to uncontrolled sewage outfalls^28^, for which the Spanish Government has been sued by the European Commission^29^. The attribution was partly based on a recent coastal study in the eastern Mediterranean Sea, that reported an increase in the abundance and N_2_ fixation activity of *Trichodesmium* after a sporadic outburst of a local wastewater treatment plant^30^. In this study the authors suggested that, due to the low N_2_ fixation activity measured, *Trichodesmium* could have shifted to a mixotrophic nutrition using wastewater-derived DOM compounds. Although the magnitude of the bloom in the Canary Islands was orders of magnitude larger in terms of density compared to that of the Mediterranean Sea^26^, we wanted to verify whether wastewater outfalls could have promoted or helped maintain *Trichodesmium* blooms in the nearshore waters of the Canary Islands. With this aim, we collected *Trichodesmium* colonies at different stages of development of the bloom (Fig. 2), and performed incubations with clear seawater and wastewater from an urban sewage outfall to the south of Gran Canaria Island and examined its impact on N_2_ and carbon fixation rates.

## Results and discussion

**Figure 3:**
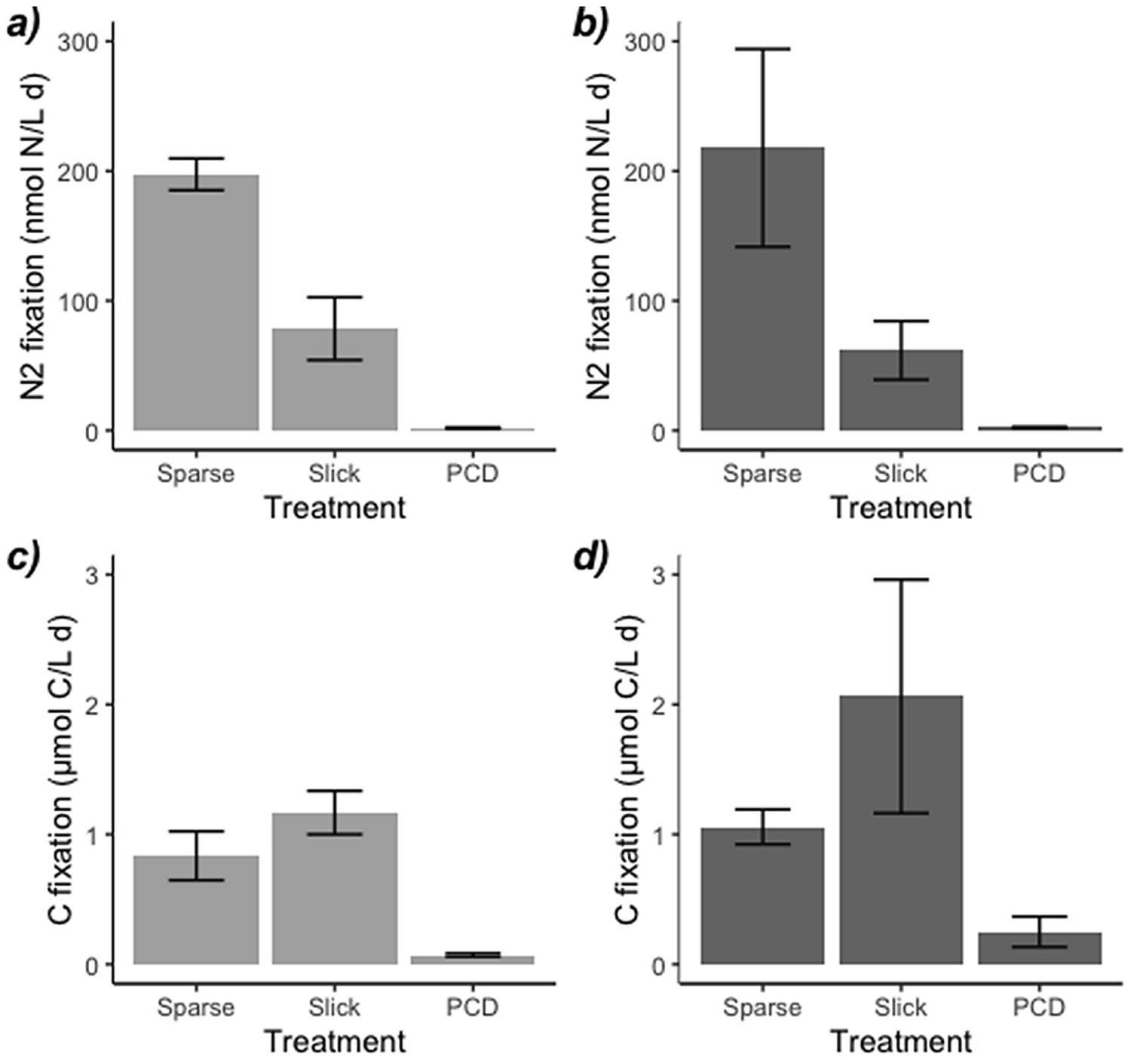
(**a**,**b**) N_2_ and (**c**,**d**) carbon fixation rates of sparse, slick and PCD-like *Trichodesmium* colonies in incubations with clear seawater (light grey) and sewage water (dark grey).

**Figure 4:**
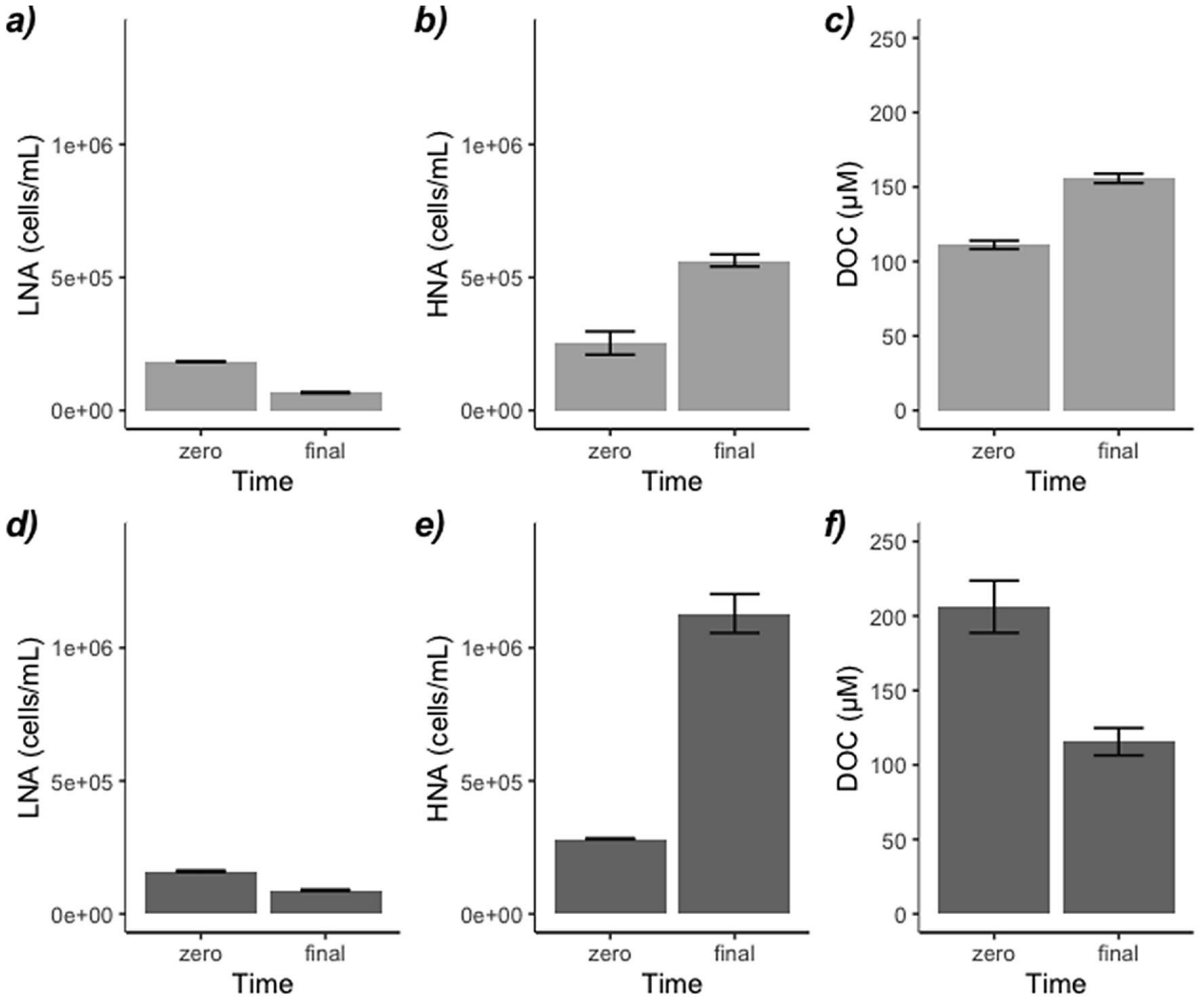
Initial and final low nucleic acid bacteria (LNA), high nucleic acid bacteria (HNA) and DOC concentrations in 24 h incubations with clear seawater (**a**–**c**, light grey) and sewage water (**d**–**f**, dark grey).

We incubated *Trichodesmium* from three different bloom development stages (sparse, slick and PCD-like, Fig. 2) with clear and sewage waters. Our experiments showed that N_2_ fixation rates in clear water incubations were not significantly different from those of sewage incubations (Fig. 3, t-test p = 0.98), which indicates that sewage did not promote N_2_ fixation in *Trichodesmium*. The concentrations of dissolved organic carbon (DOC) and nitrogen (DON) in sewage waters were double than in clear waters (Table 1), which is likely responsible for the increase in bacterial abundance in sewage waters with respect to clear waters (Table 2). In support of these results, the abundance of the most active high nucleic acid bacteria (HNA) tripled when incubated with sewage for 24 h (Fig. 4e) coinciding with a net consumption of DOC (Fig. 4f), a trend which was not observed in clear water incubations (Fig. 4b,c). *Trichodesmium* could have responded positively to DOP inputs as observed elsewhere^31,32^, but in our study DOP concentrations of clear and sewage waters were not significantly different (Table 1). We cannot exclude however, the possibility that there were toxic components in sewage waters that may have locally promoted the death of *Trichodesmium* close to the coast, as wastewaters are known to impair the metabolic activity of phytoplankton^33^.

**Table 1:** Temperature, inorganic and organic nutrient concentrations within *Trichodesmium* blooms at three different developmental stages (sparse, slick and PCD-like), as well as in clear and sewage-affected seawater.

Site	Latitude (°N)	Longitude (°E)	Temperature (°C)	NO_3_^−^ + NO^2–−^ (µM)	PO_4_^3−^ (µM)	NH_4_^+^ (µM)	DOC (µM)	DON (µM)	DOP (µM)
Sparse *Trichodesmium* colonies	27.46° 382′	15.43° 243′	24.7	0.37 ± 0.06	0.12 ± 0.01	0.47 ± 0.17	163.68 ± 15.41	2.63 ± 0.78	0.50 ± 0.02
Slick *Trichodesmium* colonies	27.48° 343′	15.44° 776′	23.64	0.27 ± 0.01	0.16 ± 0.04	0.25 ± 0.03	232.87 ± 33.95	1.51 ± 0.20	0.55 ± 0.04
PCD-like *Trichodesmium* colonies	27.44° 930′	15.41° 307′	23.75	0.28 ± 0.01	2.64 ± 0.41	0.97 ± 0.24	418.48 ± 51.74	2.35 ± 0.18	0.26 ± 0.10
Clear seawater	27.44° 420′	15.44° 547′	23.45	0.26 ± 0.08	0.15 ± 0.01	0.23 ± 0.02	112.21 ± 3.68	1.20 ± 0.28	0.48 ± 0.06
Sewage water	27.49° 37′	15.46° 78′	23.34	0.27 ± 0.04	0.16 ± 0.01	1.84 ± 2.47	218.09 ± 1.99	2.00 ± 0.68	0.40 ± 0.05

**Table 2:** Abundance of heterotrophic and autotrophic picoplankton within *Trichodesmium* blooms in three development stages (sparse, slick and PCD-like), and in clear and sewage waters.

Site	Bacteria (cells mL^−1^)	*Prochlorococcus* (cells mL^−1^)	*Synechococcus* (cells mL^−1^)	Picoeukaryotes (cells mL^−1^)
Sparse *Trichodesmium* colonies	4.3 ± 0.01 × 10^5^	8.8 ± 0.02 × 10^4^	1.3 ± 0.2 × 10^4^	2.6 ± 0.3 × 10^3^
Slick *Trichodesmium* colonies	3.0 ± 0.02 × 10^5^	4.0 ± 0.5 × 10^4^	2.0 ± 0.8 × 10^4^	2.2 ± 0.05 × 10^3^
PCD-like *Trichodesmium* colonies	4.3 ± 0.03 × 10^5^	5.4 ± 0.3 × 10^4^	3.2 ± 0.7 × 10^4^	1.2 ± 0.3 × 10^3^
Clear seawater	3.5 ± 0.06 × 10^5^	1.7 ± 0.03 × 10^4^	0.4 ± 0.05 × 10^4^	0.3 ± 0.02 × 10^3^
Sewage water	4.3 ± 0.07 × 10^5^	9.3 ± 0.4 × 10^4^	1.6 ± 0.4 × 10^4^	2.9 ± 0.07 × 10^3^

N_2_ fixation rates were ~ 200, 70 and 2 nmol N L^−1^ day^−1^ in sparse, slick and PCD samples, respectively, in both clear or sewage water incubations (Fig. 3a,b). We confidently attribute these rates to *Trichodesmium*, since our incubations included concentrated *Trichodesmium* biomass and clear or sewage water prefiltered by 20 µm (see “Methods”). Diazotrophs smaller than 20 µm such as UCYN-A have been reported in the surroundings of the Canary Islands^34^, but their N_2_ fixation activity is one to two orders of magnitude lower than that measured here. Our N_2_ fixation rates were three, two and one order of magnitude higher than those observed during a sewage outburst in the eastern Mediterranean Sea^30^. The N_2_ fixation rates of sparse colonies were at the high end of volumetric rates compiled in the global N_2_ fixation database^35^, and in the range of those observed in hotspots of diazotrophy such as the western tropical South Pacific^36^. This indicates that *Trichodesmium* were healthy and active at the time of sampling. Between June and August 2017, the Canary Islands experienced optimal conditions for the development of *Trichodesmium*: unusually warm seawater temperatures (> 23 °C), enhanced stratification of the water column and several dust deposition peaks superimposed on a lower but steady dust supply throughout the summer months^27^. There was no evidence that *Trichodesmium* consumed DOC and DON despite their higher concentrations in sewage waters than in clear waters (Table 1). This agrees with previous studies that point towards a mixotrophic nutrition in *Trichodesmium* only when inorganic nutrients are not available^24,26^.

The N_2_ fixation rates of the three bloom development stages were significantly different (one-way ANOVA p = 1.5 × 10^–5^ and p = 0.003 for clear and sewage waters, respectively). This sequential decrease in N_2_ fixation between bloom development stages agrees well with those observed in culture and field experiments^19,20,37,38^, and is clearly depicted by an increase in the release of PO_4_^3−^, NH_4_^+^ and DOC as cells die and the bloom decays (Table 1).

Carbon fixation rates were not significantly different between clear and sewage water incubations either (t-test, p = 0.07). Contrary to N_2_ fixation rates, carbon fixation was 1.4 and ~ 2 times higher in slick samples than in sparse stages in clear and sewage water incubations, respectively (Fig. 3c,d). However, this enhancement was only statistically significant in clear water incubations (one-way ANOVA p = 0.0002). Carbon fixation is performed by the bulk phytoplanktonic community and not only by *Trichodesmium,* however, the abundance of picophytoplankton in slick samples was not higher than that observed in the other bloom stages (Table 2).

The recurrent PCD-like status of the *Trichodesmium* accumulations observed along the Canary Islands’ coasts in the summer of 2017 (Fig. 1) further reinforce our hypothesis that sewage outfalls were not causing bloom reactivation and raises the question of whether they were, on the contrary, toxic to *Trichodesmium*. We however note that the chemical composition of sewage waters may be highly variable depending on their source (e.g. urban vs. rural domestic sewage, hotel resorts, golf courses, desalinization plants, etc.), potentially leading to differences in their impact on bacteria, non N_2_ fixing phytoplankton and diazotrophs.

In conclusion, our results support the hypothesis that sewage outfalls south of the islands did not trigger the formation of new blooms, nor sustain or enhance blooms originated previously offshore. Although this study examines only the potential effects of sewage waters on the growth of *Trichodesmium*, other results (which will form the basis of a complementary paper) provide evidence that the 2017 bloom originated in open ocean waters of a large part of the Canary Current region, and the colonies were transported towards the coasts by the regional circulation and local winds. The progression from sparse colonies in open ocean waters, followed by surface slicks nearer the islands to finally decaying blooms accumulated in the lee of the islands strongly supports this hypothesis^27^.

Trends in ocean temperature in the past two decades^39^ indicate that the surface waters of the Canary Current are warming unabatedly, presumably favoring the more common appearance of *Trichodesmium* blooms in the near future. This has unknown consequences for the Canary region, as blooms have also occurred in the years 2018–2020, although with less intensity than in 2017. In order to predict the development of these blooms it is therefore necessary to determine which factors (or combination of factors) trigger and maintain the blooms from their origin to their collapse. The results of this study shed some light on this issue by clearly showing that coastal sewage outfalls do not enhance or maintain these blooms near the coast.

## Methods

### *Carbon and N*_*2*_* fixation measurements*

In order to test the effects of sewage waters on the N_2_ and carbon fixation activity of *Trichodesmium*, we sampled colonies from near surface waters south of Gran Canaria Island in September 2017 (Table 1). To evaluate the behavior of colonies experiencing different developmental stages, we sampled waters with *Trichodesmium* under three different bloom development phases (Fig. 2): (i) sparse colonies -’sparse’-, (ii) colonies accumulated in the surface as slicks-’slick’-, and (iii) collapsing accumulated colonies -’PCD-like’-, collected at three different locations south of Gran Canaria Island (Table 1). Clear seawater devoid of *Trichodesmium* colonies was sampled further offshore and sewage water was collected from a coastal outlet off Puerto Rico village (27° 47′ 17″ N 15° 42′ 40″ W, Table 1). In situ temperature was measured using a Hydrolab LH4 probe.

Sparse, slick and PCD-like colonies were concentrated using a 20 µm mesh sieve. Subsequently, 5 mL of the concentrate was distributed in triplicate acid-washed 2.3 L polycarbonate bottles (Nalgene) containing either clear seawater or sewage water from separate coastal areas (Table 1), previously filtered through 20 µm mesh to remove predators. Each bottle was spiked with 2.5 mL ^15^N_2_ (98 atom %, Euriso-top) and ^13^C-labeled bicarbonate (NaH^13^CO_3_; ≥ 98 atom %, Sigma Aldrich, 10 atom % final enrichment) as previously described^40^. The bottles were incubated in shaded incubators with surface seawater for 24 h. At the end of incubations, the content of the bottles was filtered through pre-combusted (5 h, 450 °C) GF/F filters (Whatman) and stored at – 20 °C until analysis. The concentration of particulate nitrogen and carbon as well as the isotopic ratio of samples (^15^N/^14^N and ^13^C/^12^C) were obtained by means of a Thermo Flash 1112 elemental analyzer interfaced by a Conflo III with a Thermo Delta V Advantage isotope ratio mass spectrometer. To ensure an accurate calculation of N_2_ fixation rates, background dissolved ^15^N atom % enrichments were determined in all incubations by membrane inlet mass spectrometry as previously described^41^.

### Nutrient and dissolved organic matter concentrations

Seawater samples for the analysis of inorganic nutrients and dissolved organic nitrogen and phosphorus (DON and DOP, respectively) were collected from clear waters, sewage-affected waters and within the *Trichodesmium* blooms (sparse, slick and PCD-like, see above). Samples for the determination of dissolved organic carbon (DOC) concentrations were collected from within *Trichodesmium* blooms as above, but also at the start and end of incubations for carbon and N_2_ fixation measurements. All samples were filtered through pre-combusted GF/F filters (Whatman) before storage at – 20 °C.

Samples for the analysis of nitrate and nitrite (NO_3_^−^ + NO_2_^−^), phosphate (PO_4_^3−^) and ammonium (NH_4_^+^) were collected in 15 mL polyethylene tubes and stored at − 20 °C. Nutrient concentrations were determined using a Technicon II segmented-flow autoanalyzer. Samples for DOC analyses were collected in HCl-washed 20 mL polycarbonate tubes (Nalgene), stored at – 20 °C, and DOC concentrations determined with a TOC-V Shimadzu as detailed in Santana-Falcón et al.^42^. DON and DOP concentrations were collected in 50 mL polyethylene tubes and analyzed by the wet oxidation of total dissolved nitrogen and phosphorus (TDN and TDP, respectively), with subsequent subtraction of NO_3_^−^ + NO_2_^−^ and PO_4_^3−^ concentrations, respectively using standard wet oxidation methods^43^.

### Autotrophic and heterotrophic picoplankton

Abundances of autotrophic (*Prochlorococcus* and *Synechococcus* type cyanobacteria and pigmented picoeukaryotes) and heterotrophic prokaryote assemblages were determined by flow cytometry. Samples (1.6 mL) were preserved with paraformaldehyde (2% final concentration), left 15 min at 4 °C in the dark to fix, deep frozen in liquid nitrogen and stored at – 80 °C until analyzed. Fixed samples were thawed, stained in the dark for a few minutes with a DMS-diluted SYTO-13 (Molecular Probes Inc.) stock (10:1) at 2.5 µM final concentration, and run through a BD FACSCalibur cytometer with a laser emitting at 488 nm. High and Low Nucleic Acid content prokaryotes (HNA, LNA) were identified in bivariate scatter plots of side scatter (SSC-H) versus green fluorescence (FL1-H). Autotrophic picoplankton were discriminated in plots of orange fluorescence (FL2) versus red fluorescence (FL3) and picocyanobacteria (*Prochlorococcus* and *Synechococcus*) were subtracted from HNA prokaryote counts. Samples were run at low or medium speed until 10.000 events were captured. A suspension of yellow–green 1 µm latex beads (10^5^–10^6^ beads ml^−1^) was added as an internal standard (Polysciences, Inc.).
